# The roadmap for community engagement: co-design and evaluation of a toolkit to strengthen community–academic research partnerships

**DOI:** 10.1093/pubmed/fdag039

**Published:** 2026-06-07

**Authors:** Emily M D’Agostino, Christoph P Hornik, Isa Granados, Janet Kasper, Al Richmond, Allyn Daman, Princess Abbot-Grimes, Camille Brown-Lowery, Jeannine Sato, Tatiana Vizcaíno, Claudia Corchado, Mildred Council, Minerva Freeman, Camille Griggs, Ann F Huggins, Wes Gray, Amanda Sparling, Allison Swart, Cody D Neshteruk

**Affiliations:** Duke Clinical Research Institute, Duke University School of Medicine, 2400 Pratt St, Durham, NC 27705, USA; Department of Population Health Sciences, Duke University School of Medicine, 215 Morris St, Durham, NC 27701, USA; Department of Orthopedic Surgery, Duke University School of Medicine, 311 Trent Drive, Durham, NC 27710, USA; Duke Global Health Institute, Duke University, 310 Trent Drive, Durham, NC 27710, USA; Duke Clinical Research Institute, Duke University School of Medicine, 2400 Pratt St, Durham, NC 27705, USA; Department of Pediatrics, Duke University School of Medicine, 2301 Erwin Road, Durham, NC 27705, USA; Department of Population Health Sciences, Duke University School of Medicine, 215 Morris St, Durham, NC 27701, USA; Nemours Children’s Health, 807 Children's Way, Jacksonville, FL 32207, USA; United Way of Merced County, 547 W Main St, Merced, CA 95340, USA; Community-Campus Partnerships for Health (CCPH), PO Box 12124, Raleigh, NC 27605, USA; Duke Clinical Research Institute, Duke University School of Medicine, 2400 Pratt St, Durham, NC 27705, USA; Duke Clinical Research Institute, Duke University School of Medicine, 2400 Pratt St, Durham, NC 27705, USA; Duke Clinical Research Institute, Duke University School of Medicine, 2400 Pratt St, Durham, NC 27705, USA; Duke Clinical Research Institute, Duke University School of Medicine, 2400 Pratt St, Durham, NC 27705, USA; Community-Campus Partnerships for Health (CCPH), PO Box 12124, Raleigh, NC 27605, USA; United Way of Merced County, 547 W Main St, Merced, CA 95340, USA; Cultiva Central Valley, 3341 M St, Merced, CA 95348, USA; Pitt County All-Stars 4-H Club, 403 Government Circle, Greenville, NC 27834, USA; Pitt County Development Corporation, Inc, 3725 NC Highway 222, Fountain, NC 27829, USA; Bethel Advocacy Center, 2197 Old River Road, Greenville, NC 27834, USA; Pitt County Board of Public Health, 201 Government Circle, Greenville, NC 27834, USA; Pitt County NAACP, 800 W 5th St, Greenville, NC 27834, USA; Pitt County Health Department, 201 Government Cir, Greenville, NC 27834, USA; Pitt County Health Department, 201 Government Cir, Greenville, NC 27834, USA; Pitt County Health Department, 201 Government Cir, Greenville, NC 27834, USA; Duke Clinical Research Institute, Duke University School of Medicine, 2400 Pratt St, Durham, NC 27705, USA; Department of Population Health Sciences, Duke University School of Medicine, 215 Morris St, Durham, NC 27701, USA; Department of Pediatrics, Duke University School of Medicine, 2301 Erwin Road, Durham, NC 27705, USA

**Keywords:** community engagement, community-academic partnerships, community-based participatory research, community-engaged research, health equity

## Abstract

**Background:**

The Roadmap for Community Engagement (Roadmap) is a publicly online available resource co-designed with community partners that aims to support effective research engagement. The Roadmap is designed to be accessible and useful to a wide range of community members, public health practitioners, researchers, and community health advocates. The current study aimed to assess the Roadmap’s perceived usefulness, relevance, and potential for implementation.

**Methods:**

Data were collected over 12 months using a mixed methods design. Partner surveys, focus groups, and field notes were collected from biweekly academic-community partner collaboration sessions, two Roadmap content-review cycles, and a 2-day evaluation event with community partners. Data were analyzed separately and combined using a concurrent narrative format.

**Results:**

Among 90 community partners, 94% reported the Roadmap was useful, 92% anticipated strengthened connections, and 89% agreed it addressed organizational needs. Qualitative themes highlighted accessibility, adaptability, and feasibility for sustaining partnerships as strengths of the Roadmap. Potential challenges included language access, limited infrastructure to implement the Roadmap, financial and time constraints.

**Conclusions:**

The Roadmap is a rigorously co-developed, adaptable toolkit that can support equitable research partnerships. Findings justify formal effectiveness testing and implementation studies on scalability across settings to advance health equity.

## Background

Public health efforts to reduce chronic disease inequities depend on research that is generated with, rather than for, communities. Yet authentic community–academic research partnerships remain difficult to achieve in many settings because of persistent distrust, limited organizational research capacity, and misaligned priorities between stakeholders.^1–3^ These conditions are most acute in communities that stand to benefit the most from participatory research, where historical harms and deficit-based narratives have eroded confidence in academic institutions and constrained collaboration.^1–3^

Community-engaged research (CER) frameworks and community-based participatory research (CBPR) principles offer guidance on shared decision-making, transparency, and capacity building.^4–7^ However, many widely cited models articulate “what” should be done at a values or principles level while offering fewer concrete, stepwise resources that help community-based organizations (CBOs) operationalize the “how” of engagement (planning, staffing, budgeting, communications, data collection, reporting, and sustainability). Although prior work in Community Partnered Participatory Research (CPPR) has addressed the process of partnership with community engagement,^8–11^ partnerships often rely on one-off trainings for community partners or ad hoc tools that are difficult to replicate, evaluate, or adapt across contexts.^12^^,^^13^ The gap is particularly evident for smaller CBOs that have deep community trust but limited infrastructure to meet research requirements for compliance, documentation, and data quality.

To address these implementation barriers, our team of community partners and academic researchers proposed pairing practical tools with facilitation from a large, trusted community organization that builds capacity across a local network. In this model, an experienced “anchor” organization mentors smaller CBOs, provides technical assistance, and brokers relationships with academic teams and public health professionals, thereby lowering the risk of participation while protecting community priorities.^1–3^ In this model, the anchor partner typically has well-established relationships and collaborations with academic and public health organizations, often acting as a liaison and advocate for smaller CBOs, such as elevating community voices to promote equity in research, identifying research questions that are meaningful to community members, and ensuring that financial support is provided to support ongoing collaborations and sustain projects. The anchor concept emerged from prior large-scale initiatives in which community partners emphasized the need for a locally trusted intermediary to coordinate logistics, align expectations, and support equitable power-sharing.^2^^,^^3^^,^^14^

Building on this rationale, our team of community partners and academics co-developed the Roadmap for Community Engagement (Roadmap) over the last four years (2021–25; https://youandmehealthy.org/roadmap/). The Roadmap was developed as part of the You & Me COVID Free study, which examined strategies to promote timely and equitable access to COVID-19 testing in support of decision-making around safe public health practices.^15^ The Roadmap is a publicly available toolkit to foster effective research engagement and is designed to be accessible and useful to a wide range of community members, public health practitioners, researchers, and community health advocates. The Roadmap provides staged guidance and ready-to-use materials across five domains identified by community partners as essential for effective research engagement: community engagement, program planning, communications, operations, and research reporting.^16^ The Roadmap also adopts the tiered anchor-partner approach to support community partner mentorship, horizontal collaboration among CBOs, and accountability for equitable practices.

This study aims to examine perceived usefulness, relevance, and implementation potential of the Roadmap to inform subsequent effectiveness testing and scale-up. We describe the multi-phase co-design process with an anchor organization and a broad CBO network, detail the components and facilitation model, and present mixed-methods findings from partner bi-weekly collaborative sessions, partner content reviews of the Roadmap, and a 2-day evaluation event held with study partners and community members in Merced, California (CA). Together, these features aim to move from principle to practice by offering an approach and a set of tools that enable community organizations to adopt, lead, and adapt the Roadmap content to their goals and unique context to support research engagement.

## Methods

### Study design

We conducted a multi-phase approach to iteratively co-design and co-evaluate the Roadmap with community partners between 2021 and 2025. The design included sequential development and refinement phases: (i) initial Roadmap development during the You & Me COVID Free study (2021)^15^; (ii) application during You & Me: Test and Treat (YMTT), 2022–24^16^; and (iii) adaptation and refinement through biweekly co-design sessions, structured content reviews, and a countywide evaluation event (2025). This paper reports on Phase 3, a mixed-methods evaluation of perceived usefulness, relevance, and implementation potential. The Duke University Health System Institutional Review Board provided ethics approval for this study (PRO00112479).

### Setting and participants

Participants were based in Merced County, CA, and Pitt County, NC. Both settings comprise areas with racially and ethnically diverse populations and high burdens of chronic disease and socioeconomic disadvantage.^17^^,^^18^ We partnered with the United Way of Merced County (UWMC) and Pitt County Health Department (PCHD) as the anchor organizations. We engaged 90 community members and 47 CBOs, including social service agencies, advocacy groups, schools, emergency service providers, and grassroots organizations (Table 2). Participants included representatives from anchor, community, and academic partners who contributed to co-design meetings, surveys, and evaluation events.

### Roadmap background and development

The Roadmap builds on prior large-scale, community-engaged public health initiatives that paired capacity-building with trusted, locally led communication strategies to promote equitable access to health resources.^14^^,^^15^ The toolkit utilized a novel, tiered model of CER^5^ that worked with anchor partners, large community organizations with established trusted relationships with smaller, local CBOs, to promote trust, relevance, and shared goals. This approach ensured contextually relevant research and provided structured mentorship to strengthen CBO research capacity.

**Figure 1:**
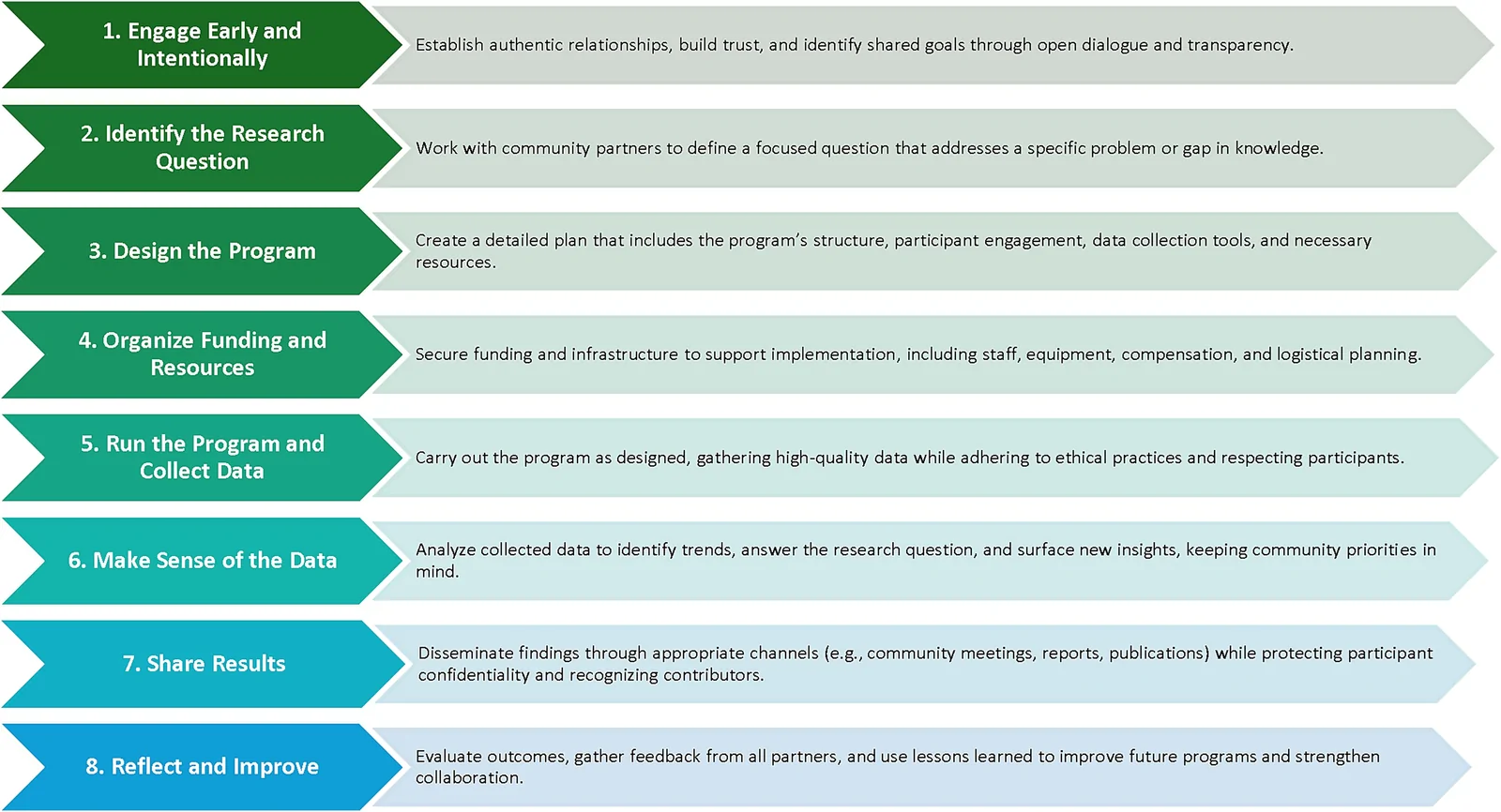
Roadmap core components.

The toolkit was co-designed with community partners in Merced County and Pitt County to guide community members and CBOs in planning and developing health-related programs and initiatives through community-engaged partnership and in support of health equity. Its primary purpose was to offer community-based strategies for research engagement, program planning, implementation, and sustainability through user-friendly tools and templates that break down the process into actionable steps. Building on this tiered anchor-partner model, the Roadmap now provides staged guidance and ready-to-use materials across five domains (community engagement, program planning, communications, operations, and research reporting) organized into eight core components (Fig. 1), as determined in our prior analysis of the partnership lessons learned using the Roadmap.^19^

### Initial application: You & Me: test and treat

The toolkit was applied in the YMTT study (2022–24)^16^ to strengthen community-academic research partnerships. Anchor partners (UWMC and PCHD) and CBOs co-designed project objectives, protocols, and dissemination of findings. Between February 2023 and June 2024, 841 participants enrolled (see Supplementary Table 1). Most were adults (82%) and resided in Merced County, CA (81%) or Pitt County, NC (6%), identified as White (40%), Black/African American (36%), and Hispanic/Latino (40%). English (75%) was the primary language. Participants reported high satisfaction with the COVID-19 test-kit program, 71% rated satisfaction ≥7 on a 10-point scale, and 20,000 COVID-19 test kits were distributed across both counties, primarily through anchor and community partners. In total, the project engaged two anchor partners from both counties and >10 CBOs that co-led local dissemination and outreach activities. YMTT project strengths, partnership lessons learned, and the toolkit effectiveness were then examined using a concurrent triangulation approach to inform necessary adaptations.^1^^,^^6^^,^^7^ Strengths identified included strong communication and mutual respect, but also highlighted the need for improved sustainability planning and accessible tools. Critical lessons learned across four primary themes emerged when engaging community organizations in research: Communication; Collaboration; Technical Infrastructure; and Sustainability and Leadership (see Table 1). Next steps to foster effective research partnerships utilizing the toolkit included prioritizing trust, accessibility, communication, sustainability, customizability, and equity.^19^

**Table 1:** Themes identified across four content-review cycles (*n* = 12 unique reviewers; April–august 2025).

Barrier identified by community partners	Relevant Roadmap components	Roadmap approaches to address the barrier
Language barriers	1, 5	Engage early with multilingual leaders; plan for interpretation and translation; collect data in preferred languages.
Time constraints	1, 5	Schedule around community availability; build trust by respecting working hours; use flexible data collection methods.
Limited availability of interpreters during late hours	1, 4	Plan logistics in advance; budget for off-hours compensation or alternative interpretation methods.
Limited awareness of research goals	1, 2	Establish open dialogue, clarify goals and benefits, co-develop the research question.
Mistrust or hesitation to share personal info	1, 5, 7	Build trust over time, ensure ethical protections, and communicate clearly how data will be used and protected.
Need for childcare	4, 5	Include funding for childcare or provide it during events/data collection.
Lack of incentives or unclear motivation to participate	4, 5	Co-design appropriate, culturally respectful incentives (not always financial); emphasize community impact.
Use of unfamiliar technology	3, 5	Select accessible tools, test usability with community members, offer tech support as needed.
Difficulty contacting organizations	1	Build relationships with anchor institutions and intermediaries who can facilitate introductions.
Community fatigue/too many small deadlines	3, 5	Build a realistic timeline with buffer periods; honor community pace.
Limited institutional or political support for CE	4, 7	Use results to advocate for greater CE funding; elevate community voices in dissemination.
Lack of representation from all subgroups	1, 3	Ensure inclusive planning and outreach across all subgroups. Use community leaders as connectors.
Trauma-informed engagement needs	1, 5	Train staff in trauma-informed practices; create safe and respectful environments for engagement.
Misunderstanding of policy topics	2, 6, 7	Build shared understanding through co-learning sessions and accessible dissemination of findings.

**Table 2:** Characteristics of participants and organizations at the Roadmap evaluation event (Merced County, CA; *n* = 90).

Sample characteristic	Total (*n* = 90)	%
**Language**		
English speakers	64	81
Spanish speakers	26	19
**Organization type** ^**a**^		
Nonprofits/CBOs	32	36
Government agencies	14	16
Health-related organizations	9	10
Education & youth-oriented	15	17
Faith/civic groups	4	4
Volunteer/individual efforts	4	4
Uncategorized	12	13
**Evaluation outcomes**		
Attendees completing evaluation	46	51
Respondents currently engaged or with future plans to conduct research	20	22
Reported increased understanding of role in CER	41	45
Reported involvement in CER activities	13	14

Note: percentages may not total 100% due to rounding. ^a^Total organizations represented = 47.

### Adaptation and refinement

In response to these lessons learned and identified next steps, the program engaged in an intensive 12-month toolkit adaptation and refinement period in 2025, together with community partners in both Merced and Pitt Counties. The toolkit, retitled by the community partners the Roadmap for Community Engagement, was iteratively adapted and refined across bi-weekly collaboration sessions with partners (*n* = 20 attendees) in response to Roadmap content reviews, and resulting in a co-designed toolkit to strengthen community–academic research partnerships.

### Data collection

Bi-weekly partner collaboration sessions (January–July 2025), four content-review cycles (April–August 2025), and evaluation surveys were used to refine the Roadmap.

From January to July 2025, community and academic partners met bi-weekly for 60-minute co-design sessions co-facilitated by anchor and academic leads. Sessions focused on reviewing Roadmap drafts, identifying implementation barriers, and prioritizing revisions. Agendas, notes, and transcripts were analyzed using rapid content analysis to capture key decisions and action items.

From April to August 2025, four sequential content-review cycles assessed the clarity, accessibility, and community relevance across five Roadmap sections (Introduction, Planning for Lasting Impact, Tools and Templates, Building the Program Framework, and Launching the Program). Reviewers from anchor organizations, county agencies, and CBOs from Merced and Pitt Counties provided written feedback on structure, readability, visual design, cultural and linguistic responsiveness, missing content, templates, and implementation feasibility. Feedback was synthesized, discussed in collaboration sessions, and incorporated into iterative revisions, forming one continuous co-design process.

In May 2025, UWMC and academic partners hosted a two-day evaluation event in Merced County, CA, for partners across both counties. The event included plenary sessions, small-group application activities, and facilitated networking. Participants completed surveys assessing perceived usefulness, relevance, alignment with organizational needs, and potential to strengthen partnerships.

### Data analysis

Qualitative data from bi-weekly sessions and content reviews were analyzed using a rapid content analysis, a pragmatic method for timely synthesis in implementation science research.^20–22^ Transcripts and field notes were coded deductively using implementation science constructs (acceptability, appropriateness, feasibility)^23^ and refined through team consensus to identify key themes and adaptations. Data were triangulated across field notes and partner surveys. Qualitative survey responses were summarized descriptively. Results were reported using a concurrent narrative to integrate qualitative and quantitative findings.^24^

## Results

### Bi-weekly collaboration sessions

Bi-weekly collaboration sessions included 19 participants representing community (*n* = 11), anchor (*n* = 2), and academic partners (*n* = 6). Rapid analysis of open-text responses and discussion notes indicated seven primary adaptations to strengthen the Roadmap:

1. Language access and inclusion: Incorporate budgeting guidance for professional translation and interpretation, clarify distinctions between consecutive and simultaneous interpretation, and emphasize cultural competence beyond linguistic accuracy.
2. Framing and values: Revise the Introduction to foreground trust, justice, and capacity building; explicitly recognize Community Health Workers; and clarify that the Roadmap applies to both medical and non-medical programs.
3. Document navigation and structure: Elevate the Glossary to the front matter, cross-reference key terms, and consistently label “Key Phases.”
4. Practicality and usability: Add concrete examples, shorter-term timelines, exemplar materials such as a partnership proposal, and brief “how-to” notes for advanced tools such as Strengths, Weaknesses, Opportunities, and Threats (SWOT).
5. Terminology consistency: Standardize definitions for stakeholders and personally identifiable information, remove duplications, and align template titles with narrative text.
6. Action sequencing: Create a clearer “what to do first” pathway to reduce cognitive load for new organizations.
7. Sustainability and measurement: Integrate quarterly benchmarks, role assignments, data-capture practices, and strategies to maintain engagement when urgency wanes.

Partners described these collaboration meetings as critical for establishing trust, surfacing operational barriers, and ensuring that adaptations to the Roadmap reflected real-world community and organizational capacity. The insights generated through these sessions directly informed the structured content reviews that followed.

### Community partner content reviews

A total of 12 unique reviewers participated across all four content-review cycles, representing anchor organizations, county government, and smaller CBOs from Merced and Pitt Counties. Individual reviews included eight reviewers in April, four in June, eight in July, and six in August, each assessing specific Roadmap sections and corresponding tools and templates: Glossary, Introduction, and Sections 1–2; Section 3; Section 4; and Sections 5–6.

Each review used standardized open-ended questions (e.g. “Does the phrasing work? If you have any suggestions for changes in phrasing or words that we use in this section, please describe those changes below.”) and section-specific prompts addressing clarity, visuals, and feasibility of implementation. Partners provided detailed written feedback on phrasing, organization, tone, and accessibility, and reviewed whether examples and templates were practical for real-world application.

Rapid analysis of open-text responses and discussion notes confirmed and expanded on the adaptations identified during earlier collaboration sessions. Reviewers emphasized the need for explicit guidance on language access and cultural inclusion, refinement of terminology, and more accessible navigation. One reviewer suggested, “Develop a customizable budget planning scale…include language services,” while another noted, “We should add definitions for translation and oral interpretation to make language access clearer.”

Partners also encouraged greater visual coherence and inclusion of diverse images, clearer stepwise guidance for new users, and reduction of technical jargon. As one participant wrote, “The visuals are perfect…easy to engage with and easy on the eyes,” while another recommended, ‘Add something visual to break up the text.’

Feedback from later rounds (July–August) focused on the practical usability of the Roadmap, encouraging inclusion of completed sample templates, case examples, and checklists clarifying sequence of activities. Reviewers noted the value of workflow tools, such as “a community partner onboarding checklist to show what steps come first” and suggestions to “highlight tools and templates in each section so it’s easier to navigate.”

Finally, participants highlighted both financial and partnership sustainability along with measurement as ongoing priorities, encouraging the addition of quarterly benchmarks, role assignments, and strategies to sustain engagement after initial implementation. Comments included, “Address long-term partnership and sustainability beyond funding,” and “Include examples on capacity building…like how to access or apply for funding.”

Across all four review rounds, partners described the Roadmap as clear, adaptable, and increasingly practical, noting that successive revisions enhanced both usability and relevance across a range of community-based settings.

### County-wide evaluation event

A total of 90 individuals registered for the two-day evaluation event, representing 47 unique community organizations across Merced and Pitt Counties. Among registrants, 26 (19%) identified Spanish as their preferred language. Sectors represented by participating organizations (non–mutually exclusive) included nonprofits or CBOs (*n* = 32; 36%), government agencies (*n* = 14; 16%), education and youth-oriented organizations (*n* = 15; 17%), health-related organizations (*n* = 9; 10%), faith or civic groups (*n* = 4; 4%), volunteer or individual efforts (*n* = 4; 4%), and uncategorized organizations (*n* = 12; 13%). Forty-six individuals attended the event and completed the evaluation survey, representing 51% of registered participants.

Among those who completed the evaluation (*n* = 46), 22% indicated a clear interest in conducting CER, with 19 missing responses for that item. Forty-one percent reported that the event increased their understanding of their role in community-engaged public health research, with no missing responses. Thirteen percent indicated involvement in CER activities, with no missing responses reported for that item.

## Discussion

### Main finding of this study

The Roadmap, co-designed with an anchor organization and a broad CBO network, was perceived as a useful, relevant, and adaptable toolkit for strengthening community–academic research partnerships. Across the evaluation event and content reviews, participants consistently rated the Roadmap as likely to strengthen research partnerships. Findings underscored the value of the tiered anchor model for trust and capacity building in support of equitable partnerships in research, while specifying actionable improvements in language access, documenting structure and consistency, and providing practical exemplars and both financial and program sustainability tools and metrics. These adaptations have been incorporated into the current version of the Roadmap (https://youandmehealthy.org/roadmap/) and define priorities for subsequent effectiveness testing and scale-up.^25^

### What is already known on this topic

Community engagement in research is widely recognized as essential for improving relevance, sustainability, and equity of public health interventions.^1^^,^^3^ Frameworks such as CBPR articulate core principles of engagement, including transparency, power sharing, and mutual benefit.^4^^,^^7^ CPPR provides specific guidelines as to processes to support community partnership in research,^8–11^ although most approaches focus on high-level values rather than step-by-step processes, and few provide accessible resources tailored to smaller CBOs with limited infrastructure. Prior work has also underscored the value of intermediary or “anchor” organizations in bridging academic and community priorities, but operational models are limited.^3^^,^^11^

### What this study adds

This study advances the evidence base in three ways. First, it documents a multi-year, iterative co-design process in which community and academic partners jointly developed, tested, and refined a Roadmap in support of equitable partnership in research. Both survey and qualitative data indicated strong potential for the Roadmap to support trust-building and capacity development. Second, this study introduces a tiered anchor model, integral to the Roadmap, that combines structured tools with organizational mentorship by a trusted and well-established larger organization to reduce barriers to CBO participation and promote community partner equity in the research process. Third, this study presents mixed-methods findings from community participants and CBOs, demonstrating high perceived utility and clarifying barriers related to infrastructure, sustainability, and inclusivity. These contributions provide evidence for the Roadmap as an effective toolkit for promoting robust CER partnerships.

### Limitations of this study

This study relied on self-report measures of perceived usefulness and relevance rather than objective indicators of partnership quality or downstream health outcomes. Participants were drawn from only two counties, which may limit generalizability. There was overlap between some CBOs involved in both the co-development and evaluation phases of the Roadmap, which may have introduced positive response bias. Finally, data were collected during an early stage of Roadmap implementation; the Roadmap has not yet been tested in a controlled comparative design or across diverse geographic and cultural settings.

### Implications for practice and research

Community organizations and public health professionals can use the Roadmap to guide partnership formation, clarify roles, and strengthen research engagement processes. The anchor-partner model appears promising for building capacity and trust, along with providing an avenue for enhancing financial and partnership sustainability, particularly among smaller organizations. In this study, we have examined the Roadmap’s perceived usefulness, relevance, and implementation considerations. Next steps for research include a randomized trial comparing the Roadmap intervention (anchor partner + online toolkit) compared to the online toolkit only, to evaluate use, partnership quality, equity outcomes, and cost. Future adaptations should prioritize language accessibility, culturally tailored examples, and explicit sustainability planning.

## Conclusion

The Roadmap represents a rigorously co-developed, accessible, and adaptable toolkit that operationalizes CER principles into practice. Early evaluation suggests strong perceived utility, relevance, and potential for successful implementation among diverse CBOs, with clear directions for further refinement and testing. By embedding step-by-step resources and a tiered anchor-partner model, the Roadmap has the potential to advance equitable, sustainable community–academic research partnerships that are critical for addressing chronic disease inequities. The next step is to test the Roadmap with a diverse group of community partners to assess effectiveness across partner types and contexts, and document adaptations in Roadmap utilization. Successful evaluation of the Roadmap will provide evidence for a broadly disseminatable toolkit to increase community engagement in research.

## Supplementary Material

Supplemental_Table_1_fdag039

## Data Availability

All data relevant to our findings are included in the manuscript. Further requests on the dataset shall be arranged based on a reasonable request to the corresponding author.

## Notes

**Emily M. D’Agostino**, DPH, MS, MEd, MA

**Christoph P. Hornik**, MD, PhD, MPH

**Isa Granados**, PhD, MS

**Janet Kasper**, BA

**Al Richmond**, MSW

**Allyn Daman**, MA

**Princess Abbot-Grimes**, MPH

**Camille Brown-Lowery**, MA

**Jeannine Sato**, MA

**Tatiana Vizcaíno**, MA

**Claudia Corchado**, BA

**Mildred Council**, BEd

**Minerva Freeman**, MA

**Camille Griggs**, BA

**Ann F. Huggins**, MSE, CAPM

**Wes Gray**, MPH, MAS

**Amanda Sparling**, MS

**Allison Swart**, MPH

**Cody D. Neshteruk**, PhD
