# Supplementary material for: The roadmap for community engagement: co-design and evaluation of a toolkit to strengthen community–academic research partnerships
Source: J Public Health (Oxf). 2026 Jun 7;48(3):745–53. doi: 10.1093/pubmed/fdag039 (PMC13531720; doi:10.1093/pubmed/fdag039)
Supplement: Supplementary file 1 [file supplemental_table_1_fdag039.docx]

**Table 1. Characteristics of Participants, Community Partners, and Program Implementation: You & Me: Test and Treat (YMTT) Study, 2023–2024**

| **Characteristic** | **n (%) or Description** |
| --- | --- |
| Participants (n = 841) |  |
| Adults (18+) | 690 (82) |
| Minors (8–17) | 151 (18) |
| Geographic distribution |  |
| Merced County, CA | 679 (80.7) |
| Other CA counties | 30 (3.6) |
| Pitt County, NC | 50 (5.9) |
| Other NC counties | 5 (0.6) |
| Race |  |
| White | 336 (40) |
| Black/African American | 303 (36) |
| Asian | 25 (3) |
| Native Hawaiian/Pacific Islander | 8 (1) |
| American Indian/Alaska Native | 8 (1) |
| Multiracial/Other | 59 (7) |
| Did not specify | 101 (12) |
| Ethnicity (Hispanic, Latino, or Spanish origin) |  |
| Yes | 336 (40) |
| No | 479 (57) |
| Did not specify | 25 (3) |
| Language |  |
| English | 631 (75) |
| Spanish | 109 (13) |
| Did not specify | 101 (12) |
| Recruitment and Engagement |  |
| Community org/bulletin board | 664 (79) |
| Word of mouth | 67 (8) |
| Flyer | 50 (6) |
| Social media | 34 (4) |
| Combination | 8 (1) |
| Other/unspecified | 17 (2) |
| Test kit satisfaction (1–10 scale) |  |
| Very satisfied (9–10) | 530 (63) |
| 7–8 | 67 (8) |
| 5–6 | 42 (5) |
| 3–4 | 8 (1) |
| 1–2 | 8 (1) |
| Did not rate | 185 (22) |

Note: Percentages may not total 100 due to rounding. Adults defined as participants aged ≥18 years. “Did not specify” reflects missing or unreported data. Total n = 841 participants enrolled between February 2023 and June 2024.
